# Genome-Wide Association Analysis Identifies Resistance Loci for Bacterial Leaf Streak Resistance in Rice (*Oryza sativa* L.)

**DOI:** 10.3390/plants9121673

**Published:** 2020-11-29

**Authors:** Wannapa Sattayachiti, Samart Wanchana, Siwaret Arikit, Phakchana Nubankoh, Sujin Patarapuwadol, Apichart Vanavichit, Clive T. Darwell, Theerayut Toojinda

**Affiliations:** 1 Plant Breeding Program, Faculty of Agriculture at Kamphaeng Saen, Kesetsart University, Nakhon Pathom 73140, Thailand; sattayachiti65@gmail.com; 2 National Center for Genetic Engineering and Biotechnology (BIOTEC), 113 Thailand Science Park, Pahonyothin Road, Khlong Nueng, Khlong Luang, PathumThani 12120, Thailand; samart.wan@biotec.or.th (S.W.); p.nubankoh@gmail.com (P.N.); cliveterence.dar@biotec.or.th (C.T.D.); 3 Department of Agronomy, Faculty of Agriculture at Kamphaeng Saen, Kasetsart University, Kamphaeng Saen Campus, Nakhon Pathom 73140, Thailand; siwaret.a@ku.th (S.A.); vanavichit@gmail.com (A.V.); 4 Rice Science Center, Kasetsart University, Kamphaeng Saen, Nakhon Pathom 73140, Thailand; 5 Center of Excellence on Rice Precision Breeding for Food Security, Quality, and Nutrition, Kasetsart University, Kamphaeng Saen, Nakhon Pathom 73140, Thailand; 6 Department of Plant Pathology, Faculty of Agriculture at Kamphaeng Saen, Kasetsart University, Kamphaeng Saen Campus, Nakhon Pathom 73140, Thailand; agrsujp@ku.ac.th

**Keywords:** bacterial leaf streak disease, *Xanthomonas oryzae* pv. *oryzicola* (*Xoc*), rice, genome-wide association (GWAS), broad-spectrum resistance

## Abstract

Bacterial leaf streak (BLS) caused by *Xanthomonas oryzae* pv. *oryzicola* (*Xoc*) is one of the most devastating diseases in rice production areas, especially in humid tropical and subtropical zones throughout Asia and worldwide. A genome-wide association study (GWAS) analysis conducted on a collection of 236 diverse rice accessions, mainly *indica* varieties, identified 12 quantitative trait loci (QTLs) on chromosomes 1, 2, 3, 4, 5, 8, 9 and 11, conferring resistance to five representative isolates of Thai *Xoc*. Of these, five QTLs conferred resistance to more than one *Xoc* isolates. Two QTLs, *qBLS5.1* and *qBLS2.3,* were considered promising QTLs for broad-spectrum resistance to BLS. The *xa5* gene was proposed as a potential candidate gene for *qBLS5.1* and three genes, encoding pectinesterase inhibitor *(OsPEI)*, eukaryotic zinc-binding protein (*OsRAR1)*, and NDP epimerase function, were proposed as candidate genes for *qBLS2.3*. Results from this study provide an insight into the potential QTLs and candidate genes for BLS resistance in rice. The recessive *xa5* gene is suggested as a potential candidate for strong influence on broad-spectrum resistance and as a focal target in rice breeding programs for BLS resistance.

## 1. Introduction

Rice is the staple food of more than half the world population. Rice production is threatened by several factors including abiotic stresses, such as drought, soil salinity and flooding, and biotic stresses, such as insect pests and diseases caused by pathogens. Bacterial leaf streak (BLS) disease caused by *Xanthomonas oryzae* pv. *oryzicola* (*Xoc*) is one of the most devastating diseases in rice production areas, particularly in tropical and subtropical humid zones in Asia and other regions [1]. Yield losses between 10–20% have been recorded for BLS depending on rice varieties and environmental conditions [2]. BLS, along with other diseases, is becoming of greater concern due to ongoing climate change, especially in Asia and Africa where its intensity and frequency of effect is growing. A key sustainability goal for controlling BLS is the development of resistant rice varieties, especially as the majority of rice cultivars in Asia and Africa are susceptible to the disease [3]. Therefore, it is necessary to identify resistance genes/quantitative trait loci (QTLs) and the functional background of BLS resistance in rice breeding programs for rice variety improvement.

BLS resistance is considered a quantitatively inherited trait [4]. At least 13 QTLs for BLS resistance have been mapped using classical QTL mapping approaches [4,5,6]. Among these QTLs, the *qBlsr5a* on the short arm of chromosome 5 has the largest effect in explaining variation in BLS resistance among identified QTLs (Tang et al., 2000). A potential candidate gene for *qBlsr5a* was suggested to be the gene encoding the gamma chain of transcription initiation factor IIA (TFIIAγ) or *xa5* [6]. Recently, eleven additional QTLs for broad-spectrum resistance to BLS, as well as bacterial leaf blight (BLB), have been reported using a genome-wide association study (GWAS) performed on a multiparent advanced generation intercross (MAGIC) population [7].

As with other plant disease resistance, BLS resistance genes or QTLs are probably strain-specific [7]. To successfully control the disease in a particular geographical region, therefore, it is important to identify the genes/QTLs that confer resistance to the strains and combinations of strains occurring in that region. To date, QTLs for BLS resistance have primarily been identified using Asian *Xoc* strains, notably from the Philippines and China, and alongside some African strains [4,5,6,7]. The genes or QTLs associated with the resistance to BLS caused by Thai *Xoc* strains have not been elucidated.

Genome-wide association studies (GWAS) using a large number of single nucleotide polymorphisms (SNPs) are popularly used to identify genomic regions associated for a variety of traits in plants, such as grain yield components in sorghum [8], husk tightness in maize [9] and insect resistance in soybean [10]. Recently, GWAS has been successfully used to identify QTLs for the two major rice diseases: blast [11,12,13] and bacterial blight [14,15]. GWAS has also been used to detect QTLs for BLS caused by *Xanthomonas campestris* pv. *translucens* in wheat (*Triticum aestivum* L.) based on winter wheat accessions [16], and in rice (*Oryza sativa* L.) based on a MAGIC population [7]. In this study, a GWAS analysis was performed on a diverse germplasm panel, consisting mainly of Thai rice varieties (predominantly *indica*) and some international cultivars, to identify genomic regions associated with BLS resistance against five representative isolates of Thai *Xoc*. Additionally, loci and candidate genes conferring broad-spectrum resistance to BLS were proposed.

## 2. Results

### 2.1. BLS Disease Resistance in the Rice Germplasm Collection

**Figure 1:**
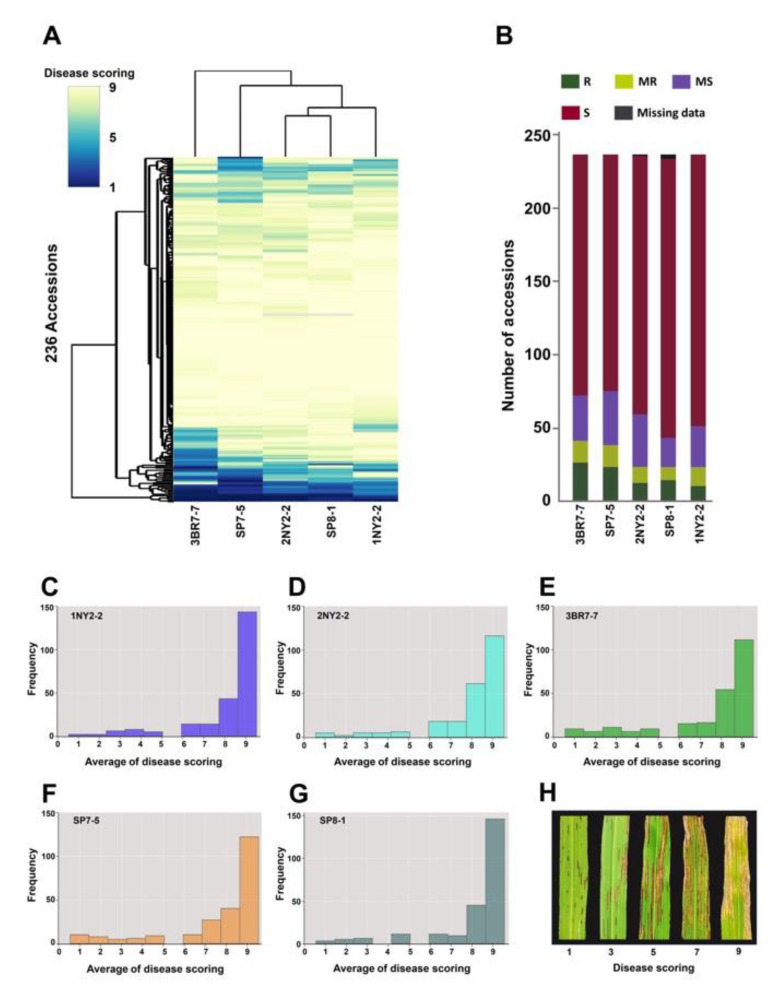
BLS disease reactions and scores of 236 rice accessions inoculated with representative isolates of Thai *Xoc*. (**A**) Hierarchical cluster of accessions and isolates based on BLS scores. (**B**) Number of accessions in R (scores of 1–3), MR (scores of 4–5), MS (scores of 5–7) and S (scores of 8–9) reactions. Distribution of the average BLS disease scores of 236 rice associations: (C–G): (**C**) 1NY2-2 isolate, (**D**) 2NY2-2 isolate, (**E**) 3BR7-7 isolate, (**F**) SP7-5 isolate and (**G**) SP8-1 isolate. (**H**) The scale 1–9 of disease scoring on infected leaves, (1 = resistant, 9 = susceptible).

We assessed bacterial leaf streak (BLS) disease resistance in 236 rice accessions (Table S1) against five representative isolates of Thai *Xoc*: 1NY2-2, 2NY2-2, 3BR7-7, SP7-5, and SP8-1 (Table S2). Based on the BLS disease scores calculated using unweighted pair-groups with arithmetic mean (UPGMA), the five *Xoc* isolates were divided into three groups: Group 1 consisted of 3BR7-7; Group 2 consisted of SP7-5, and Group 3 consisted of 2NY2-2, SP8-1, and 1NY2-2 (Figure 1a). The proportions of resistance (R) varieties to total varieties for Group 1 and Group 2 were higher than those for Group 3 (Figure 1b). For each *Xoc* isolate, the distribution of BLS severity ratings among the 236 accessions indicated that the majority of rice varieties were susceptible to these *Xoc* isolates (Figure 1c–g). Among these rice accessions, 11 varieties (4.6%) exhibited high resistance to all five *Xoc* isolates. These included five South Asian varieties: Dular (*aus*), DV85 (*aus*), Dharia (*aus*), Nona Bokra (*indica*), and Kalubala Vee (*aus*); three International Rice Research Institute (IRRI) varieties: IR4563-52-1-3-6, IR64, and IR62266; a Thai landrace, Dawk Pa-yawm; and two Thai improved varieties: San Pah-tawng 1 and RD21 (Figure S1). A number of rice varieties also exhibited isolate-specific resistance as they were resistant to between 1 and 4 *Xoc* isolates (Figure S2; Table S3).

### 2.2. Genome-Wide Association Analysis

**Figure 2:**
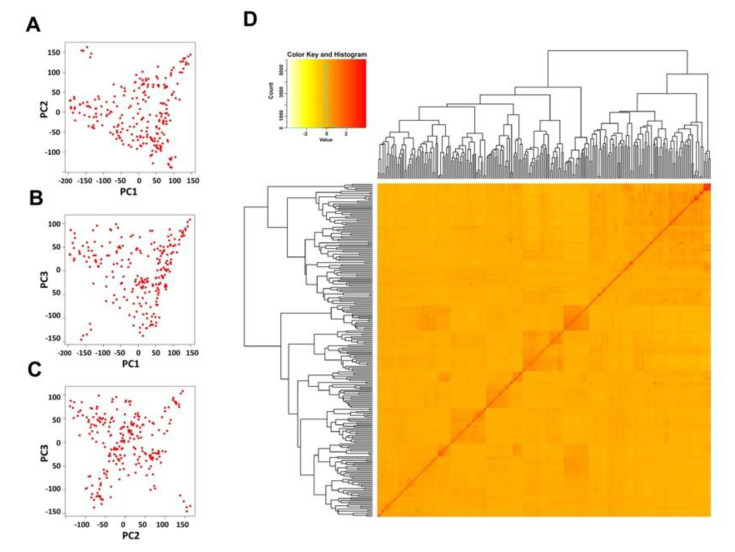
Principal component analysis (PCA) and kinship relatedness analysis of 236 genotypes. (**A**–**C**) Variation of the first three principal components (PCs): (**A**) PC1 vs. PC2, (**B**) PC1 vs. PC3 (**B**), and PC2 vs. PC3 (**C**). (**D**) Kinship matrix of 236 individuals displayed as a heatmap.

To identify genomic regions associated with rice BLS resistance, we performed a genome-wide association analysis (GWAS) in the 236 rice accessions. A total of 176,820 single-nucleotide polymorphisms (SNPs) were used as the genotype data; the average number of SNPs per chromosome was 14,735 SNPs, and the average distance between adjacent SNPs was 2249 bp (Table S4; Figure S3). At all positions SNPs had a minor allele frequency (MAF) greater than 0.05. A set of linkage disequilibrium (LD)-pruned SNPs (6422 SNPs) were used to generate kinship matrix and perform a Principal Component Analysis (PCA) in order to investigate the population structure and cryptic relationship among the 236 rice accessions. The results of the PCA and kinship analyses revealed some degree of population structure in this rice diversity panel (Figure 2). The majority of rice accessions in the diversity panel, which were landraces and improved varieties from Thailand, could be classified into a large *indica* group. However, a subgroup containing five *aus* varieties: DV85, Dular, Kalubala Vee, FR13A, and Dharia, was clearly separated from the rest (Figure 2). The result of STRUCTURE analysis based on the same set of 6422 SNPs revealed six subgroups in this diversity panel (Figure S4).

**Figure 3:**
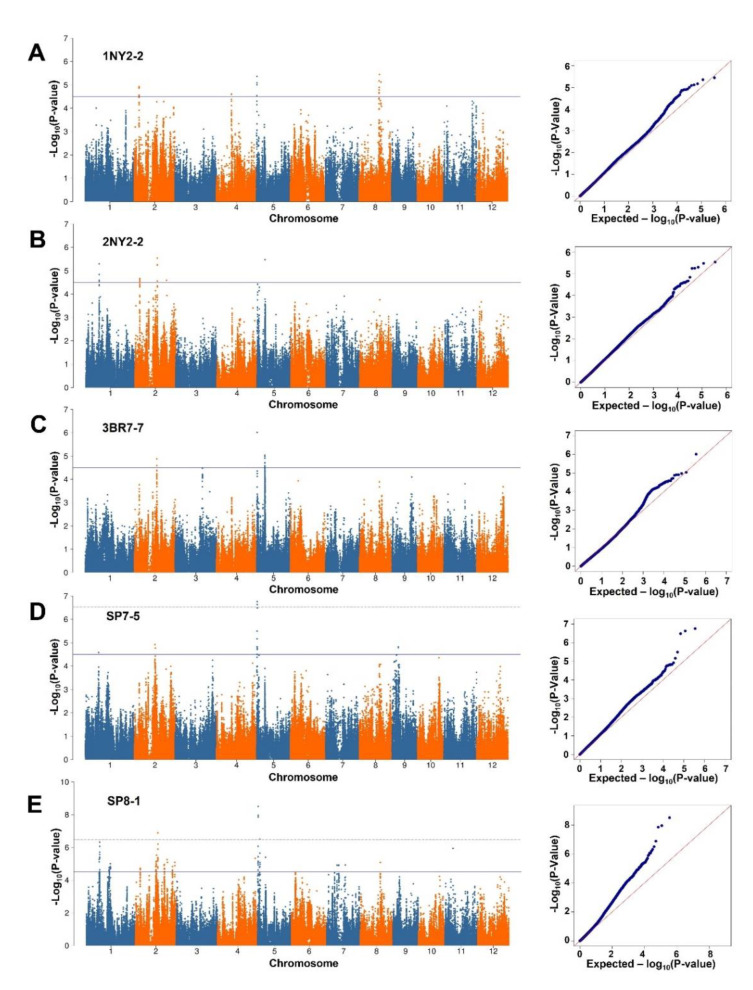
Manhattan and quantile-quantile plots resulting from the genome-wide association study (GWAS) results for BLS disease against five *Xoc* isolates: (**A**) 1NY2-2, (**B**) 2NY2-2, (**C**) 3BR7-7, (**D**) SP7-5 and (**E**) SP8-1. The cut-off threshold at −log10 (*p*) of 4.5 is indicated by black line and the Bonferroni threshold is indicated by dotted line.

The GWAS analysis, featuring a Mixed Linear Model (MLM) and incorporating the first three PCs and the kinship matrix, detected 22 regions associated with BLS resistance (Figure 3; Table 1). These regions were located on chromosomes 1, 2, 3, 4, 5, 8, 9 and 11. Heritability (h^2^), as defined by the ratio of genetic variance to the total variance, for BLS resistance according to each *Xoc* isolate was 54.28%, 49.25%, 84.42%, 52.37%, and 48.90%, for 1NY2-2, 2NY2-2, 3BR7-7, SP7-5, and SP8-1, respectively (Table 1). Regions that contained adjacent associated SNPs within an LD block (r^2^ > 0.2) were combined, resulting in 12 final QTLs: *qBLS1.1*, *qBLS2.1*, *qBLS2.2*, *qBLS2.3*, *qBLS2.4*, *qBLS3.1*, *qBLS4.1*, *qBLS5.1*, *qBLS5.2*, *qBLS8.1*, *qBLS9.1* and *qBLS11.1* (Table 2). Among these, five QTLs conferred resistance to two or more *Xoc* isolates. These included *qBLS1.1* on chromosome 1; *qBLS2.1* and *qBLS2.3* on chromosome 2; and *qBLS5.1,* and *qBLS5.2* on chromosome 5. In particular, *qBLS5.1* was significantly associated with resistance to almost all five *Xoc* isolates based on a cut-off threshold of −log10 (*p*) > 4.5 whilst a –log10 (*p*) of 4.42 was recorded for 2NY2-2. The rest of the identified QTLs appear isolate-specific. These include *qBLS2.2* on chromosome 2 and *qBLS9.1* on chromosome 9, specifically associated with SP7-5 isolate; *qBLS4.1* on chromosome 4 and *qBLS8.1* on chromosome 8, specifically associated with 1NY2-2 isolate; *qBLS3.1* on chromosome 3 and *qBLS11.1* on chromosome 11, specifically associated with 3BR7-7 isolate and SP8-1 isolate (Table 2).

### 2.3. Linkage Disequilibrium (LD) Decay and Candidate Gene Identification

**Figure 4:**
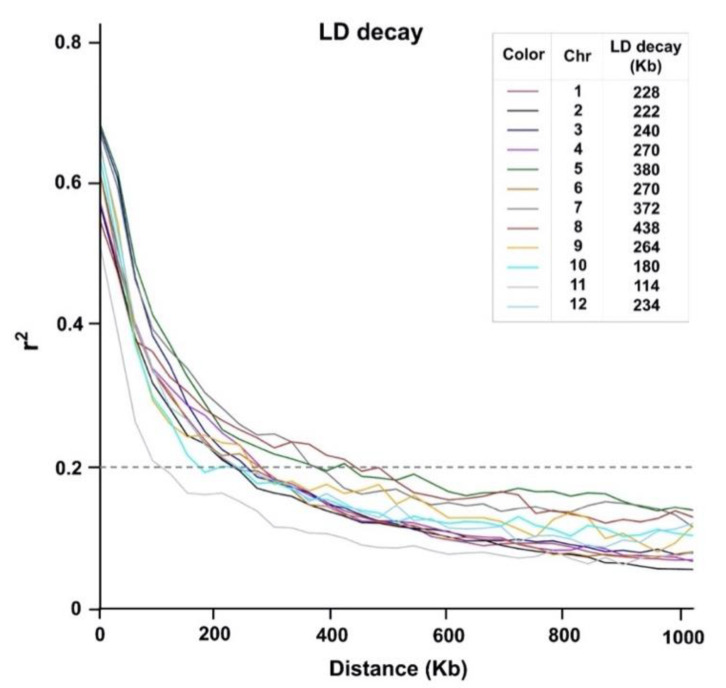
Overall chromosome-wide linkage disequilibrium (LD) decay estimated from the SNP genotypes of 236 rice associations. Each line plot represents a smoothed r^2^ for all marker pairs on each chromosome depending on the distance between marker pairs. The cut-off threshold of LD decay (r^2^ = 0.2) is indicated by a dotted line.

To determine the extent of LD decay in the diversity panel, we estimated the pairwise LD index (r^2^) based on the SNP genotype data across 12 rice chromosomes. The results show that the average LD decay was found within 268 kb (r^2^ < 0.2). The shortest distance of typical LD decay was 114 kb as identified on chromosome 11, and the longest one was 438 kb as identified on chromosome 8 (Figure 4).

We then identified candidate genes for each of the 12 QTLs by considering genes annotated within the LD block harboring the lead SNPs. The number of annotated genes for each QTL ranged from 15 to 70 genes (Table 2). Genes that contain functional SNPs, i.e., missense SNPs causing amino acid changes were primarily considered (Table 2; Table S5). These included genes that have functions related to disease resistance, such as those encoding NBS-LRR domain containing proteins: *LOC_Os01g20720*, *LOC_Os02g30150*, *LOC_Os05g12570*, *LOC_Os08g28540*, *LOC_Os08g28570*, *LOC_Os11g13410*, *LOC_Os11g13430*, *LOC_Os11g13440*; kinase family proteins: *LOC_Os01g20880, LOC_Os01g20900, LOC_Os03g43440, LOC_Os08g28710*, *LOC_Os08g28870*, and *LOC_Os08g28890*; and transcription factors: *LOC_Os02g46030*, *LOC_Os08g29660* and *LOC_Os09g10840* (Table 2).

### 2.4. Haplotype Analysis of qBLS5.1 and qBLS2.3

**Figure 5:**
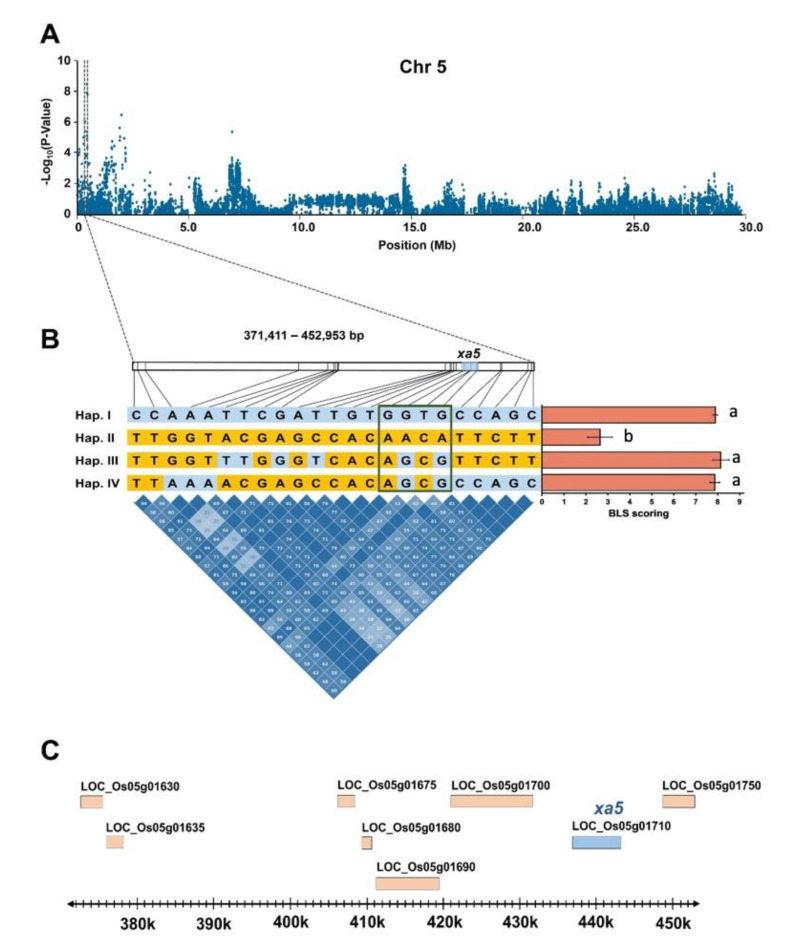
Identification of the candidate gene for the peak of *qBLS5.1* on chromosome 5. (**A**) Manhattan plot on chromosome 5 based on mixed-linear Model (MLM). X-axis shows the SNPs along the chromosome and y-axis is the –log10 (*p*) for the association. The peak region is indicated in the block between two vertical dotted lines. (**B**) Haplotype block and LD focusing on four SNPs on *xa5* gene inside the green block. The bar graph is shown the average BLS scoring. X-axis is the disease scoring scale 0–9 (0 = resistance, 9 = susceptible) and y-axis is showed the haplotype. Letters to the right most of the bar graph indicate significant groups. Color codes for nucleotides in the haplotypes: yellow represents minor alleles and blue represents major alleles. (**C**) Significant SNP position overlapping the region of *xa5* gene (*LOC_Os05g01710*).

We selected two QTLs, *qBLS5.1* and *qBLS2.3*, that we consider to be the most promising QTLs for broad-spectrum resistance to BLS caused by Thai *Xoc* isolates to further analyze haplotypes and LD structure, and to identify potential candidate genes. For *qBLS5.1*, an LD block that contained 23 associated SNPs was identified within the region between 371,411-452,953 bp. This LD block spanned eight genes, including *LOC_Os05g01710* (*Xa5*) (Figure 5a). Four haplotype patterns (Hap.I, Hap.II, Hap.III and Hap.IV) were identified within this LD block (Figure 5b; Table S6). Among these, Hap.II was associated with BLS resistance (Figure 5b; Table S7 and Figure S5). Among the 23 SNPs, four (R05000438232, R05000439566, R05000439607, and R05000440778) were found to be located in *LOC_Os05g01710*. However, they might not have a functional effect, as they are located within intronic regions (intron 3). We further examined the other two consecutive SNPs (R05000437499 and R05000437500 with the variant AG/TC), which were located in exon 1 of this gene and known to be associated with BLB resistance. These two SNPs were initially excluded from the SNP data used in the GWAS analysis because their MAF was less than 0.05. The result shows that the variant AG of this locus was associated with BLS resistance in the 236 rice accessions and correlated with Hap.II (Table S7; Figures S5 and S6).

**Figure 6:**
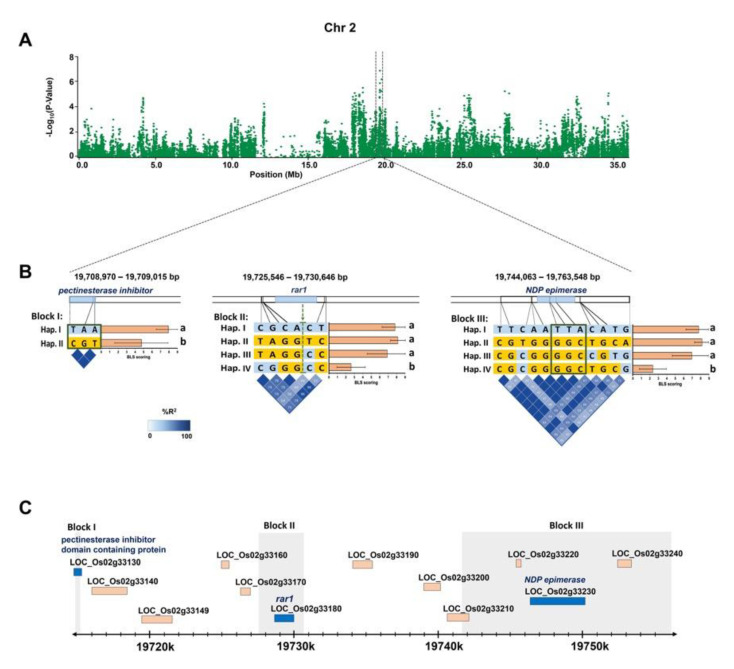
Identification of the candidate gene for the peak of *qBLS2.3* on chromosome 2. (**A**) Manhattan plot on chromosome 2 based on mixed-linear Model (MLM). The peak region is indicated in the block between two vertical dotted lines. (**B**) Haplotype blocks (upper) and LD structures (lower) of the associated SNPs. SNPs located in the genes are highlighted in the box. The bar graph shows the average BLS scores of rice accessions corresponding to each haplotype group. Letters to the right most of the bar graph indicate significant groups. BLS disease scoring ranges from 0–9 (0 = resistance, 9 = susceptible). Color codes for nucleotides in the haplotypes: yellow represents minor alleles and blue represents major alleles. (**C**) Annotated genes within the LD block region of *qBLS2.3.* The candidate gene in each LD block is highlighted in blue.

For *qBLS2.3*, three haplotype blocks of the significantly associated SNPs were identified: Block I (19,708,970–19,709,015 bp), Block II (19,725,546–19,730,646 bp), and Block III (19,744,063–19,763,548 bp) (Figure 6). Two haplotype patterns were identified in Block I, and four patterns were identified in Blocks II and III. Hap.II of Block I and the Hap.IV of Block II and III showed a significant association with the BLS resistance (Figure 6b; Figures S7–S9; Tables S6 and S8). Block I comprises three SNPs (R02019708970, R02019709012 and R02019709015) located within *LOC_Os02g33130* (encoding pectinesterase inhibitor domain containing protein). All of them were missense SNPs causing amino acid changes (Table S9). The haplotype Block II was composed of six SNPs, four of which (R02019725546, R02019725581, R02019725981 and R02019725682) were located downstream and two of which (R02019730516 and R02019730646) were located upstream of *LOC_Os02g33180* (*OsRAR1*). We further identified the variants in the *LOC_Os02g33180* and found that a SNP (R02019728136) with missense function (GAA > AAA) at the position 19,728,136 bp was associated with BLS resistance and correlated with Hap.IV in this LD block (Table S9; Figure S10). Block III composed of 12 SNPs, including the three SNPs (R02019751393, R02019751500 and R02019752412), which were located in the intronic regions of *LOC_Os02g33230* (encoding nucleotide-diphosphate-sugar epimerase). However, these three SNPs were not associated with BLS resistance (Figure 6b). We then further identified other variants in the gene. As a result, three SNPs with a missense function (R02019750786, R02019751892 and R02019751928) were identified (Table S9). Among these, R02019751892 were associated with BLS resistance and correlated with Hap.IV in this LD block (Figure S11).

We also performed a combination haplotype analysis of the two QTLs. As a result, for the *Xoc* isolates 1NY2-2 and 2NY2-2, the mean BLS disease scores of accessions that contain both superior haplotypes (the haplotypes that are associated with BLS resistance) of *qBLS5.1* and *qBLS2.3* had significantly lower values than those with only one superior haplotype of either *qBLS5.1* or *qBLS2.3* as determined by one-way analysis of variance (ANOVA). For the *Xoc* isolates 3BR7-7, SP7-5 and SP8-1, the mean BLS disease scores of accessions containing both superior haplotypes also had lower values but not significantly different from those containing a superior haplotype (Table S10). The result also clearly showed that the mean BLS disease scores of accessions without the superior haplotypes had significantly higher values for all five *Xoc* isolates.

## 3. Discussion

Bacterial leaf streak (BLS) resistance in rice has been reported as a polygenic trait. Most rice varieties cultivated in Asia and Africa are susceptible to BLS. In this study, we used 236 rice accessions, comprising Thai rice landraces and improved cultivars as well as some international varieties to identify the genomic regions associated with resistance against five representative Thai *Xoc* isolates. The distribution of BLS resistance scores indicate that the proportion of resistant accessions within our rice panel is low. This is similar to the distribution of traits in some previous GWAS analyses of major rice diseases, e.g., blast [13] and bacterial blight [14,15].

Although most of the rice varieties used for GWAS analysis in this study were categorized into the *indica* group, five *aus* varieties included in the diversity panel were clearly classified into a distinct subgroup. We used an MLM model in TASSEL that incorporated this population structure and familial relatedness in the GWAS analysis to minimize the false-positive effect that may be derived from sub-populations. According to the quantile-quantile (Q-Q) plots (Figure 3), false-positive associated SNPs could be successfully minimized in the GWAS analysis of almost all *Xoc* isolates, except for SP8-1 where the Q-Q plot showed a high inflation of -log_10_(*p*). The Bonferroni calculated threshold was more stringent (−log10 (*p*) > 6.55) but was not applied in the identification of associated SNPs in this study because only a few SNPs reached this threshold (Figure 3d,e).

*qBLS5.1* and *qBLS2.3*, were considered the most promising QTLs conferring broad-spectrum resistance to BLS in this study since *qBLS5.1* was associated with resistance to almost all five *Xoc* isolates and *qBLS2.3* was associated with the resistance to three *Xoc* isolates. *qBLS5.1* spanned 130 kb and harbored 15 genes, 13 of which contained SNPs with functional effect, i.e., missense SNPs that affect amino acid changes. *qBLS5.1* was co-localized with the *xa5* locus (*LOC_Os05g01710*), which has previously been reported as a single gene with a high effect on resistance to both BLB and BLS [6,17,18]. A lead SNP for *qBLS5.1*, R05000440778, which was identified as being associated with resistance to three *Xoc* isolates, 1NY2-2, 2NY2-2 and SP8-1, was located within this gene. The other two lead SNPs, R05000355417 and R05000466183, which were identified as being associated with resistance to 3BR7-7 and SP7-5, respectively, were also located near *xa5*. This indicates that this gene is a likely potential candidate for this QTL. *xa5* encodes mutated basal transcription factor IIA gamma subunit 5 (TFIIA*γ5*) caused by the nucleotide substitution variant (TC > AG) resulting in a change of the 39th amino acid: V39E [17]. This nucleotide substitution has previously been reported to be associated with BLB resistance [17,19]. In this study, we found that this variant was also associated with the resistance to BLS; the average disease scores of rice accessions containing the allele AG of *xa5* were significantly lower than those of the accessions carrying the TC allele across all five *Xoc* isolates (Figure S5). This result suggests that the *xa5* gene could be effectively used to control BLS in Thailand. This gene is specific to the transcription activator-like (TAL) effector encoded from both *Xoc* and *Xoo (Xanthomoas oryzae* pv. *oryzae)*. These pathogens use TAL as their primary virulence factor to promote infection by interacting with the Xa5 protein (encoded by the dominant allele of *Xa5*) and the interaction is required for the ability of TAL effectors to transcriptionally activate their targets. [20,21,22,23]. Therefore, the identified nucleotide substitutions in *xa5* (recessive allele) can decrease the ability to invade host cells of *Xoo* and *Xoc*, causing recessive loss-of-susceptibility among host plants [18,23,24]. The QTL, *qBLS2.3*, located on chromosome 2 (19.64–20.04 Mb), was not found to be overlapped by any previously reported QTLs, such as *qXO-2-1* (24.12–26.99 Mb) and *qXO-2-2* (35.29–35.78 Mb) [7]. Thus, our results suggest that *qBLS2.3* may be a novel QTL for broad-spectrum BLS resistance. There were 45 genes included in the 400 kb interval harboring *qBLS2.3*. Among these, 20 genes contain SNPs with a functional effect. Based on the haplotype analysis for this QTL, three genes, *LOC_Os02g33130* (encoding pectinesterase inhibitor domain containing protein), *LOC_Os02g33180* (*OsRAR1*), and *LOC_Os02g33230* (encoding nucleotide-diphosphate-sugar epimerase), were found to contain a specific haplotype associated with BLS resistance. Pectinesterase inhibitors have been previously implicated to play a role in defense against both bacteria [25] and fungi [26]. *OsRAR1,* interacting with *OsSGT1,* functions in basal disease resistance to rice bacterial leaf blight caused by *Xoo* and fungal blast caused by *Magnaporthe oryzae* in a race-specific resistance manner [27]. Rice genes encoding a nucleoside-diphosphate-sugar epimerase in response to pathogens has not been previously reported. However, transgenic rice plants with an overexpression of a *UDP-glucose 4-epimerase* from *Brassica rapa* have shown tolerance to bacterial blight [28]. A further study, e.g., expression analysis, is still required to verify the possible function of these genes and determine whether all or any of them confer BLS resistance in rice.

There were 11 rice accessions exhibiting high resistance to all five *Xoc* isolates (Figure S1; Table S3). Among these, six accessions contain *xa5* resistance; four of which are *aus* varieties, i.e., Dular, DV85, Kalubala Vee, Dular, Dharia, and two of which are IRRI varieties, i.e., IR4563-52-1-3-6 and IR62266. Some of these lines, such as DV85, have also been reported to contain other *Xa* genes in addition to *xa5* [29]. The rice variety IR62266 has been used as a source of broad-spectrum resistance for BLB in Thailand [30,31]. Based on our results, IR62266 could also be suggested as a donor for BLS resistance. The other five varieties that were highly resistant to all five *Xoc* isolates do not contain *xa5* conferred resistance. These include a landrace (Dawk Pa-yawm) and two improved varieties (RD21 and San Pah-tawng 1) from Thailand, an indica variety (Nona Bokra), and an IRRI variety (IR64). This suggests that other genes besides *xa5* play a role in broad-spectrum resistance to BLS in rice. These rice varieties could be used as donors in breeding programs for BLS resistance and the identification of other BLS resistance genes.

The candidate genes for other QTLs included several genes that are in the same families as the known R genes, such as genes encoding NBS-LRR domain containing proteins. The NBS-LRR or nucleotide-binding site leucine-rich repeat types genes are the largest R gene families in plants [32,33]. More than 400 genes encoding the coiled-coil nucleotide-binding site leucine-rich repeat (CC-NBS-LRR) or their partial homologs have been identified from the sequenced genomes in rice [34]. The function of LRR domains in many R proteins is thought to take part in protein-protein (elicitor–receptor) interactions [35]. Genes encoding kinase family proteins were also annotated within the identified QTL regions, such as *LOC_Os01g20880* and *LOC_Os01g20900* corresponding to *qBLS1.1* that conferred resistance to 2NY2-2, SP7-5 and SP8-1; *LOC_Os03g43440* corresponding to *qBLS3.1* that conferred resistance to 3BR7-7; *LOC_Os08g28710*, *LOC_Os08g28870*, and *LOC_Os08g28890* corresponding to *qBLS8.1*. In addition, several transcription factors were also found in several QTLs, such as *LOC_Os02g46030* (MYB family transcription factor), *LOC_Os08g29660* (WRKY69) and *LOC_Os09g10840* (transcription factor). Although these genes could be proposed as possible candidate genes for BLS resistance in rice based on our GWAS results, their specific functions in conferring BLS resistance still need to be verified. Moreover, since the pathogen relies on TAL effectors as virulence factors that bind to effector-binding elements (EBE) in the promoters of host genes, the variations in EBEs in the promoters of candidate genes in rice accessions should also be identified once the sequences of TAL effector genes in these *Xoc* isolates are available.

## 4. Materials and Methods

### 4.1. Association Mapping Panel and BLS Resistance Evaluation

Our association mapping panel consists of 236 rice accessions, including 127 accessions of Thai landraces, 77 accessions of Thai improved rice varieties, and 32 accessions of international varieties. Resistance to bacterial leaf streak (BLS) disease of rice accessions in this panel was evaluated against five *Xoc* isolates: 1NY2-2, 2NY2-2, 3BR7-7, SP7-5 and SP8-1, under greenhouse conditions at the Rice Science Center, Kasetsart University, Thailand, between August and September 2019. The five *Xoc* isolates represent four diversity groups of *Xoc* collected from different parts of Thailand. Inocula were prepared from three-day-old purified bacterial cultures growing on peptone sucrose agar (PSA) media [36]. The bacterial suspension was prepared by diluting in distilled water and adjusting the density to a working concentration of 10^8^ cfu/mL (OD_600nm_ = 0.2). Inoculation was performed in the greenhouse by spraying 200 mL of bacterial inoculum onto the leaves of 21-day old plants, which were grown in plastic trays. After inoculation, each tray was placed in a plastic box with a covered lid and incubated overnight. The tray was then taken out of the box and maintained in the greenhouse equipped with a misting system to control humidity (75% relative humidity). BLS disease evaluation was done 14 days after inoculation by using the IRRI Standard Evaluation System (SES) [37]. The experimental design was a Randomized Complete Block Design (RCBD) with three replications of four seedlings per accession.

### 4.2. Genome-Wide Association Analysis

GWAS was carried out on 176,820 SNP markers, using TASSEL (Trait Analysis by Association, Evolution and Linkage) software version 5.2.54 [38]. The SNP data used for GWAS were homozygous genotypes with a 90% or greater call rate and with a minor allele frequency (MAF) greater than 5%. These SNPs were obtained from SNP data sets derived from a whole-genome re-sequencing project at the Rice Gene Discovery, National Center for Genetic Engineering and Biotechnology, Thailand (unpublished data) and called using the Nipponbare IRGSP 1.0 rice reference genome. Linkage disequilibrium (LD) pruning was performed to obtain a subset of unlinked SNPs using a variant pruning tool (-indep-pairwise 50 10 0.2) in PLINK [39]. Principal component (PC) analysis and kinship analysis were performed using TASSEL based on the LD-pruned SNPs to obtain PC and kinship matrices. The STRUCTURE algorithm [40] was run using a model with admixture and correlated allele frequencies, with 3 independent replicates run for each genetic cluster (K) value, with K ranging from 1 to 8, using a burn-in of 10,000 steps and a run length of 10,000 Markov Chain Monte Carlo (MCMC) iterations. Ln(PD) values were derived for each K and plotted to find the plateau of the ΔK. The final population structure was calculated using structure harvester (http://taylor0.biology.ucla.edu/structureHarvester/) [41]. The mixed linear model (MLM), which incorporated a kinship matrix (K) along with the covariate principal components (P: the first three principal components), was performed. PCA plots and the kinship heatmap were generated by GAPIT [42]. The SNPs density of each chromosome was created by using the R package RIdeogram [43]. The significant associated SNPs were defined at a uniform threshold of –log_10_ (*p*-value) ≥ 4.5 (arbitrary). The manhattan plots and quantile-quantile plots for GWAS results were created using the R package qqman [44].

### 4.3. Linkage Disequilibrium (LD) Decay, Haplotype Analysis and Candidate Gene Identification

Genome-wide LD decay versus genetic distance was estimated by a pairwise analysis of adjacent SNPs within a chromosome using PopLDdecay [45]. The LD structures and haplotypes within the QTL regions were inferred based on the significantly associated SNPs using Haploview software version 4.2 [46]. To analyze the haplotype combination between *qBLS5.1* and *qBLS2.3*, we compared the mean BLS disease scores among the 236 rice accessions, which were classified into four classes: (1) those with only the superior haplotype (the haplotype that is associated with BLS resistance) of *qBLS5.1*, (2) those with only the superior haplotypes of *qBLS2.3*, (3) those with both superior haplotypes of *qBLS5.1* and *qBLS2.3,* and (4) those without any superior haplotype, using one-way ANOVA. The annotation of genes located within each QTL region was identified using the database from Rice genome annotation project (http://rice.plantbiology.msu.edu) and effects of SNP variation in the genes were identified using the Variant Effect Predictor (VEP https://asia.ensembl.org/info/docs/tools/vep/index.html).

## 5. Conclusions

The results in this study provide an insight into the potential QTLs and candidate genes for BLS resistance in rice. The GWAS analysis results revealed 12 QTLs associated with BLS resistance, five of which conferred resistance to more than one *Xoc* isolate. *xa5* identified within *qBLS5.1* is suggested to be a high potential candidate gene, and rice varieties containing *xa5* could be used as donors for broad-spectrum resistance to BLS. In addition, some of the landraces and improved varieties that exhibited high resistance to BLS but do not contain *xa5* may potentially serve as new sources of BLS resistance.

## Figures and Tables

**Table 1:** The single-nucleotide polymorphisms (SNPs) that are associated with bacterial leaf streak (BLS) resistance to five *Xoc* isolates.

Isolate	Chr.	Position (bp)	−log10 (*p*-Value)	SNP	MAF (%)	Effect	Marker R^2^	Genetic Var.	Residual Var.	Heritability (%)
1NY2-2	2	4,181,654	4.93	T/C	17.65	1.95	0.09	1.14	0.96	54.28
	4	13,430,963	4.61	C/T	10.24	−1.76	0.08			
	5	440,778	5.36	G/A	14.44	−2.95	0.10			
	8	17,854,983	5.45	A/G	6.34	2.52	0.10			
2NY2-2	1	11,593,588	5.29	C/T	6.40	−2.31	0.10	0.98	1.01	49.25
	2	4,181,736	4.66	T/C	17.78	1.92	0.09			
	2	19,690,256	5.54	C/T	12.77	2.21	0.10			
	2	27,868,581	4.59	C/T	14.66	2.03	0.08			
	5	440,778	4.42	G/A	14.44	−2.60	0.08			
	5	6,954,155	5.47	T/C	12.50	−2.32	0.10			
3BR7-7	2	19,714,517	4.88	A/G	15.22	3.38	0.09	2.60	0.48	84.42
	3	23,996,396	4.48	A/G	12.56	3.84	0.09			
	5	355,417	6.01	T/A	12.95	−5.01	0.12			
	5	7,180,089	5.04	A/G	57.45	1.55	0.09			
SP7-5	1	11,593,588	4.58	C/T	6.40	−2.68	0.08	2.10	1.91	52.37
	2	18,036,539	4.92	C/T	9.95	2.55	0.09			
	5	466,183	*6.85*	T/C	13.76	−4.59	0.13			
	9	5,885,730	4.82	G/T	22.99	1.75	0.09			
SP8-1	1	11,593,588	6.32	C/T	6.40	−2.56	0.12	1.11	1.16	48.90
	2	19,690,256	*6.88*	C/T	12.77	2.69	0.13			
	5	440,778	*8.50*	G/A	14.44	−4.09	0.17			
	11	7,331,015	5.93	C/T	11.86	2.75	0.13			

**Table 2:** Twelve regions associated with the resistance to five *Xoc* isolates based on a GWAS.

QTLs	Chr.	X_oc_ Isolates	LD Block (Mb)	No. of Loci	No. of Loci with Mi Sense/Nonsense SNPs	Genes Related with Resistance
*qBLS1.1*	1	2NY2-2, SP7-5, SP8-1	11.39–11.69	31	14	LOC_Os01g20720 (CC-NBS-LRR); LOC_Os01g20880 (OsWAK3) LOC_Os01g20900 (OsWAK4)
*qBLS2.1*	2	1NY2-2, 2NY2-2	4.17–4.37	28	16	LOC_Os02g07650 (zinc-binding protein)
*qBLS2.2*	2	SP7-5	17.88–18.21	26	13	LOC_Os02g30150 (disease resistance protein)
*qBLS2.3*	2	2NY2-2, SP8-1,3BR7-7	19.64–20.04	45	20	LOC_Os02g33130 (pectinesterase inhibitor domain containing protein); LOC_Os02g33180 (OsRAR1) LOC_Os02g33230 (nucleoside-diphosphate-sugar (NDP) epimerase LOC_Os02g33400 (OsFBL9 - F-box domain and LRR containing protein); LOC_Os02g33450 (peroxiredoxin);
*qBLS2.4*	2	2NY2-2	27.85–28.07	19	16	LOC_Os02g46030 (MYB family transcription factor);
						LOC_Os02g45850 (B3 DNA binding domain containing protein)
*qBLS3.1*	3	3BR7-7	23.93–24.26	28	12	LOC_Os03g43390 (F-box/LRR domain containing protein) LOC_Os03g43440 (CAMK includes calcium/calmodulin dependent protein kinases)
*qBLS4.1*	4	1NY2-2	13.44–13.76	18	9	LOC_Os04g23700 (lectin protein kinase family protein)
*qBLS5.1*	5	3BR7-7, 1NY2-2, SP8-1, SP7-5	0.33–0.46	15	13	LOC_Os05g01710 (transcription initiation factor IIA gamma chain)
*qBLS5.2*	5	2NY2-2, 3BR7-7	6.87–7.28	44	22	LOC_Os05g12140 (Leucine Rich Repeat family protein);
						LOC_Os05g12570 (NB-ARC domain containing protein)
*qBLS8.1*	8	1NY2-2	17.41–18.28	70	43	LOC_Os08g28540 (resistance protein LR10);
						LOC_Os08g28570 (resistance protein);
						LOC_Os08g28670 (pathogenesis-related Bet v I family protein);
						LOC_Os08g28710 (receptor protein kinase CRINKLY4 precursor);
						LOC_Os08g28870 (receptor-like protein kinase 5 precursor);
						LOC_Os08g28890 (protein kinase family protein);
						LOC_Os08g29660 (WRKY69)
*qBLS9.1*	9	SP7-5	5.86–6.19	28	14	LOC_Os09g10840 (transcription factor)
*qBLS11.1*	11	SP8-1	7.21–7.44	20	10	LOC_Os11g13410 (mla1, encoding NB-ARC domain-containing disease resistance protein) LOC_Os11g13430 (RGH1A) LOC_Os11g13440 (RGH1A)

## Supplementary Materials

The following are available online at https://www.mdpi.com/2223-7747/9/12/1673/s1, Figure S1 Rice accessions that have broad-spectrum resistance to five Xoc isolates (A: 1NY2-2, B: 2NY2-2, C: 3BR7-7, D: SP7-5, E: SP8-1), Figure S2. Rice accessions resistant to certain Xoc isolates. (A) Rice accession resistant to the 3BR7-7 isolate. (B) Rice accessions resistance to SP8-1 and SP7-5 isolates. Codes for Xoc isolates: A = 1NY2-1, B = 2NY2-2, C = 3BR7-7, D = SP7-5 and E = SP8-1, Figure S3. Density and distribution of SNPs throughout 12 rice chromosomes, Figure S4. Population structure at K = 6 for 236 rice accessions, Figure S5. Box plots of BLS disease scores of rice accessions in each haplotype of the LD block within qBLS5.1, Figure S6. Box plots of BLS disease scores of rice accessions in each group of the genotypes on xa5. (A) 1NY2-2 isolate, (B) 2NY2-2 isolate, (C) 3BR7-7 isolate, (D) SP7-5 isolate, and (E) SP8-1 isolate. Significance level is indicated by asterisks (**** *p* < 0.00001), Figure S7. Box plots of BLS disease scores of rice accessions corresponding to each haplotype of the LD Block I of qBLS2.3, Figure S8. Box plots of BLS disease scores of rice accessions corresponding to each haplotype of the LD Block II within qBLS2.3, Figure S9. Box plots of BLS disease scores of rice accessions corresponding each haplotype of the LD Block III within qBLS2.3, Figure S10. Box plots of BLS disease scores of rice accessions corresponding to the SNP genotype in LOC_Os02g33180, Figure S11. Box plots of BLS disease scores of rice accessions corresponding to the SNP genotypes in the gene *LOC_Os02g33230.* (A) The plots at position R02019750786. (B) The plots at position R02019751892. (C) The plots at position R02019751928. Table S1. Rice accessions used in the genome-wide association study, Table S2 Details on five Xoc isolates used in the study, Table S3 BLS disease scores of five Xoc isoalated in rice germplasm, Table S4 Summary of SNP numbers on 12 rice chromosomes, Table S5. Genes annotated within each QTL region, Table S6. The ecotype information and the number of varieties in each haplotype of *qBLS5.1* and *qBLS2.3,* Table S7 Statistical test for the association between haplotypes on qBLS5.1 and BLS disease scores, Table S8 Statistical test for the association between haplotypes on qBLS2.3 and BLS disease scores, Table S9. Candidate genes and annotation of SNPs with moderate and high impacts, Table S10. Combination haplotype analysis of qBLS5.1 and qBLS2.3.

## Abbreviations

GWAS	Genome-wide association study
LD	Linkage disequilibrium
PCA	Principal component analysis
MLM	Mixed Linear Model
BLS	Bacterial leaf streak
BLB	Bacterial leaf blight
Xoc	*Xanthomonas oryzae* pv. oryzicola
Xoo	*Xanthomonas oryzae* pv. oryzae
QTL	Quantitative Trait Loci
SNP	Single nucleotide polymorphism
MAF	Minor allele frequency
MAGIC	Multiparent advanced generation intercross
TAL EBE	Transcription activator-like effector Effector binding element
