# Supplementary material for: Genome-Wide Association Analysis Identifies Resistance Loci for Bacterial Leaf Streak Resistance in Rice (Oryza sativa L.)
Source: Plants (Basel). 2020 Nov 29;9(12):1673. doi: 10.3390/plants9121673 (PMC7761455; doi:10.3390/plants9121673)
Supplement: Supplementary file 1 [file plants-09-01673-s001.zip › Supplementary/Fig.S2-Rice accessions resistant to certain Xoc isolates.docx]

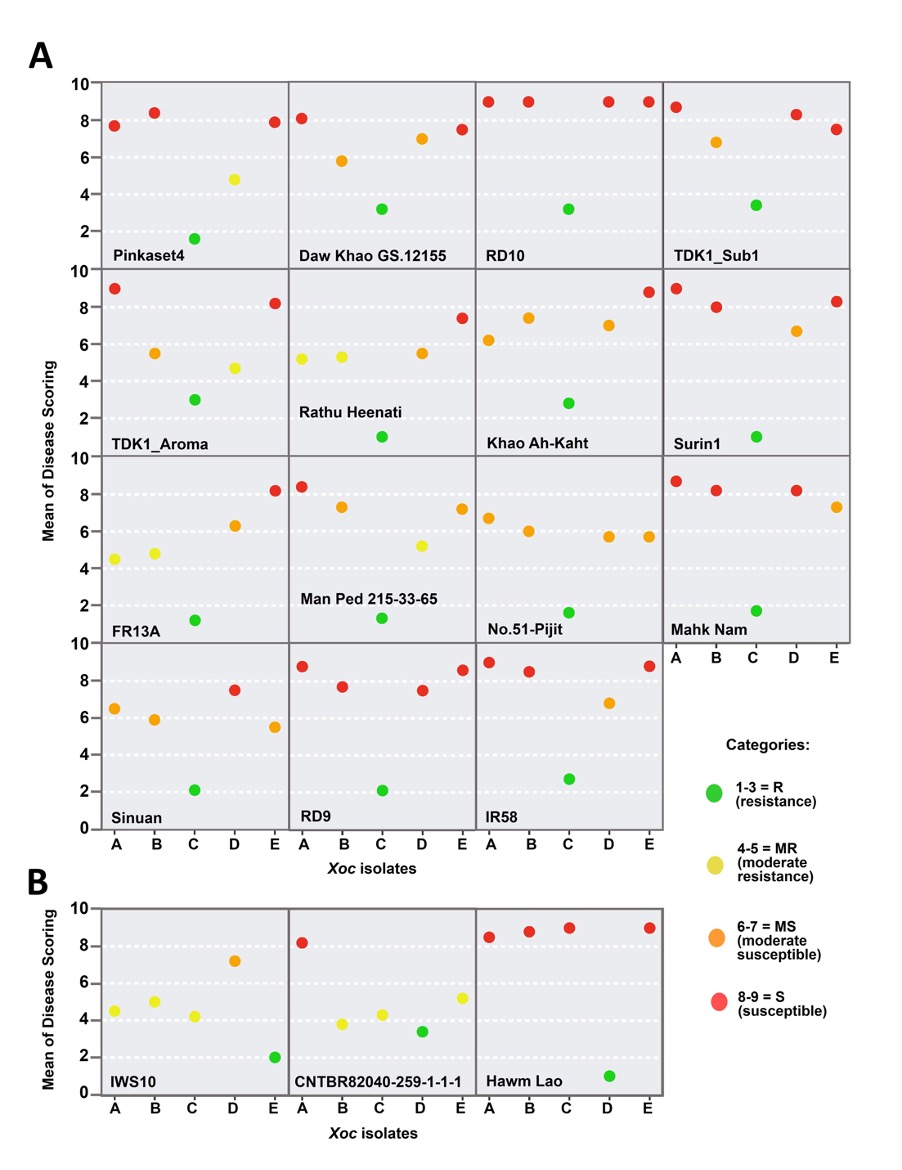


**Figure S2.** Rice accessions resistant to certain Xoc isolates. (A) Rice accession resistant to the 3BR7-7 isolate. (B) Rice accessions resistance to SP8-1 and SP7-5 isolates. Codes for Xoc isolates: A = 1NY2-1, B = 2NY2-2, C = 3BR7-7, D = SP7-5 and E = SP8-1.
