# Supplementary material for: Genome-Wide Association Analysis Identifies Resistance Loci for Bacterial Leaf Streak Resistance in Rice (Oryza sativa L.)
Source: Plants (Basel). 2020 Nov 29;9(12):1673. doi: 10.3390/plants9121673 (PMC7761455; doi:10.3390/plants9121673)
Supplement: Supplementary file 1 [file plants-09-01673-s001.zip › Supplementary/Fig.S1-resistance lines.docx]

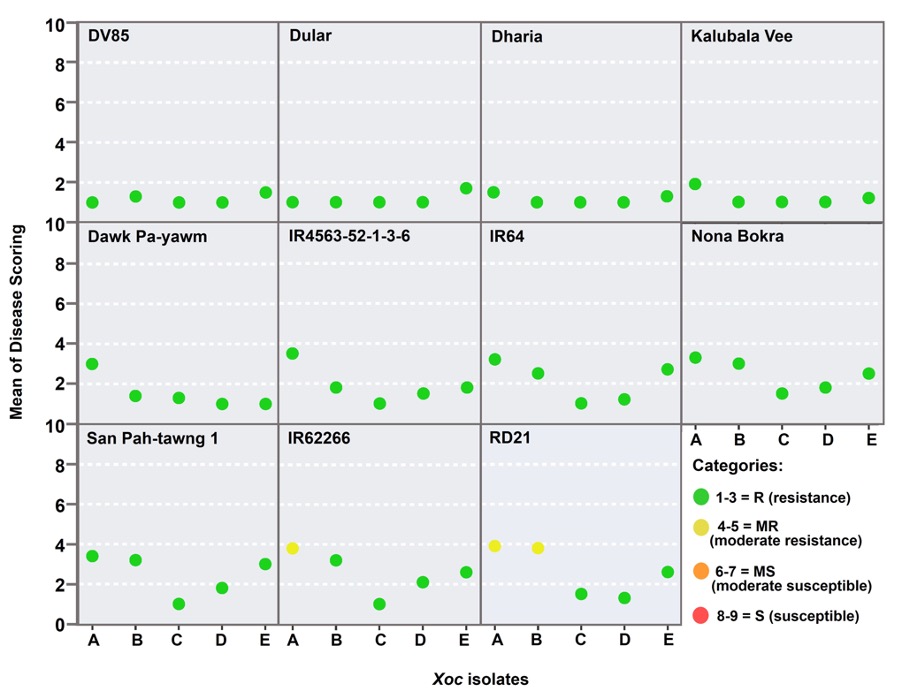


**Figure S1** Rice accessions that have broad-spectrum resistance to five Xoc isolates (A: 1NY2-2 , B: 2NY2-2, C: 3BR7-7, D: SP7-5, E: SP8-1)
