# Supplementary material for: Genome-Wide Association Analysis Identifies Resistance Loci for Bacterial Leaf Streak Resistance in Rice (Oryza sativa L.)
Source: Plants (Basel). 2020 Nov 29;9(12):1673. doi: 10.3390/plants9121673 (PMC7761455; doi:10.3390/plants9121673)
Supplement: Supplementary file 1 [file plants-09-01673-s001.zip › Supplementary/Fig.S6-box plots xa5.docx]

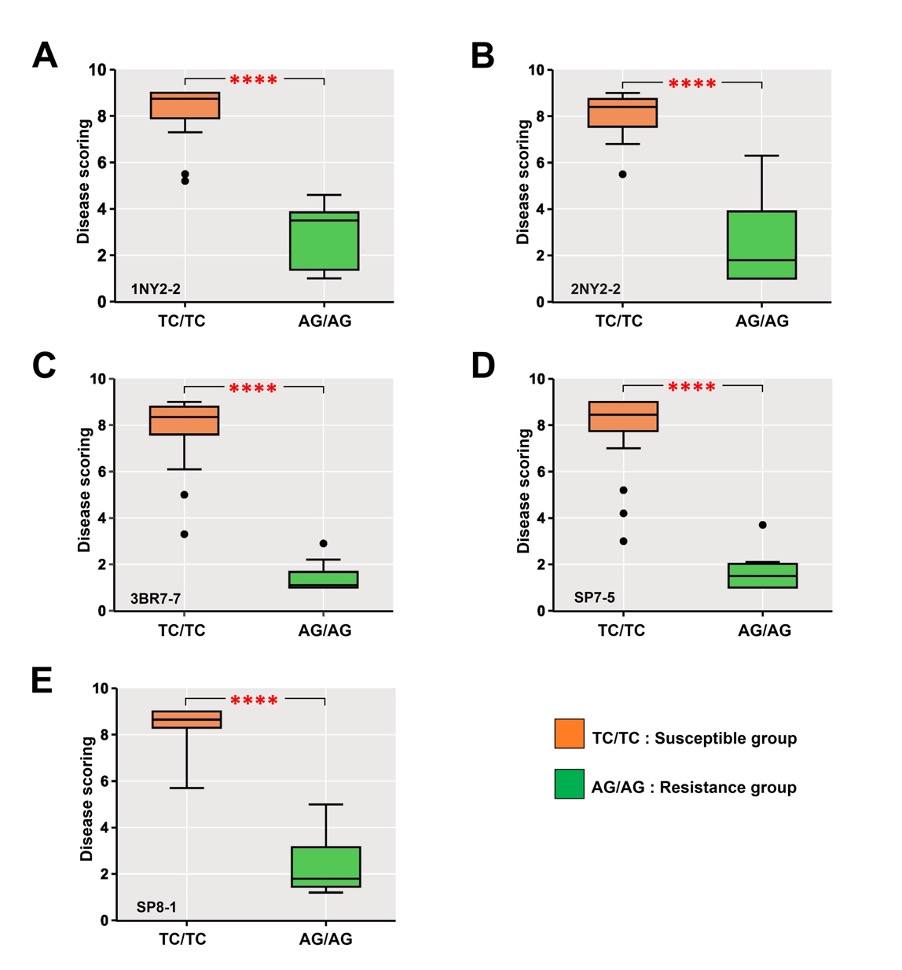


**Figure S6**. Box plots of BLS disease scores of rice accessions in each group of the genotypes on *xa5*. (A) 1NY2-2 isolate, (B) 2NY2-2 isolate, (C) 3BR7-7 isolate, (D) SP7-5 isolate, and (E) SP8-1 isolate. Significance level is indicated by asterisks (****P < 0.00001)
