# Supplementary material for: Genome-Wide Association Analysis Identifies Resistance Loci for Bacterial Leaf Streak Resistance in Rice (Oryza sativa L.)
Source: Plants (Basel). 2020 Nov 29;9(12):1673. doi: 10.3390/plants9121673 (PMC7761455; doi:10.3390/plants9121673)
Supplement: Supplementary file 1 [file plants-09-01673-s001.zip › Supplementary/Fig.S7-Box plots Block I qBLS2.3.docx]

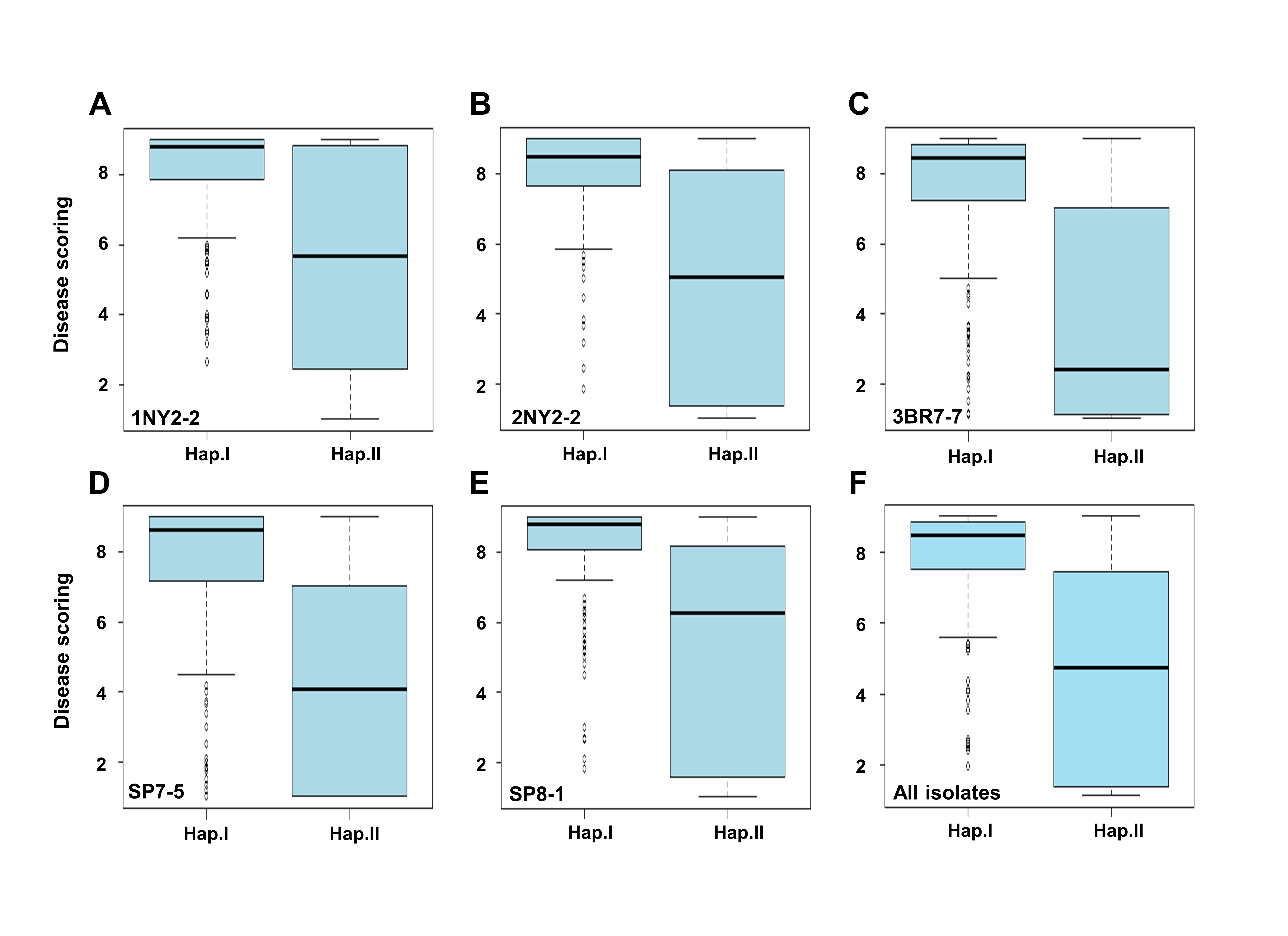


**Figure S7**. Box plots of BLS disease scores of rice accessions corresponding to each haplotype of the LD Block I of *qBLS2.3*
