# Supplementary material for: Genome-Wide Association Analysis Identifies Resistance Loci for Bacterial Leaf Streak Resistance in Rice (Oryza sativa L.)
Source: Plants (Basel). 2020 Nov 29;9(12):1673. doi: 10.3390/plants9121673 (PMC7761455; doi:10.3390/plants9121673)
Supplement: Supplementary file 1 [file plants-09-01673-s001.zip › Supplementary/Table S6-ecotypes and haplotypes.docx]

**Table S6.** The ecotype information and the number of varieties in each haplotype of *qBLS5.1* and *qBLS2.3.*

| **Haplotype** | **Ecotype** | **Number of accessions divided by origin** | | | | | | **Total number of accessions in each haplotype** |
| --- | --- | --- | --- | --- | --- | --- | --- | --- |
|  |  | **South Asia** | **IRRI** | **Lao PRD** | **Myanmar** | **Thailand** | **Unknown** |  |
| **Haplotypes of *qBLS5.1*** | | | | | | | | |
| Hap I | Improved variety | 1 | 9 | 2 | 1 | 68 | - | 211 |
|  | Landrace | 4 | - | - | 1 | 122 | 3 |  |
| Hap II | Improved variety | - | 2 | - | 1 | 2 | - | 9 |
|  | Landrace | 4 | - | - | - | - | - |  |
| Hap III | Landrace | - | - | - | - | 5 | - | 5 |
| Hap IV | Improved variety | - | 2 | - | - | 6 | - | 10 |
|  | Landrace | 2 | - | - | - | - | - |  |
| **Haplotypes of Block I of *qBLS2.3*** | | | | | | | | |
| Hap I | Improved variety | - | 12 | 2 | 1 | 68 | - | 208 |
|  | Landrace | 1 | - | - | - | 121 | 3 |  |
| Hap II | Improved variety | - | - | - | 1 | 3 | - | 12 |
|  | Landrace | 6 | - | - | 1 | 1 | - |  |
| **Haplotypes of Block II of *qBLS2.3*** | | | | | | | | |
| Hap I | Improved variety | 1 | 13 | 1 | 1 | 66 | - | 187 |
|  | Landrace | - | - | - | - | 102 | 3 |  |
| Hap II | Improved variety | - | - | 1 | - | 3 | - | 20 |
|  | Landrace | 1 | - | - | - | 15 | - |  |
| Hap III | Improved variety | - | - | - | - | 3 | - | 12 |
|  | Landrace | 3 | - | - | 1 | 5 | - |  |
| Hap IV | Improved variety | - | - | - | - | 1 | - | 6 |
|  | Landrace | 4 | - | - | - | 1 | - |  |
| **Haplotypes of Block III of *qBLS2.3*** | | | | | | | | |
| Hap I | Improved variety | 1 | 13 | 1 | 1 | 68 | - | 192 |
|  | Landrace | - | - | - | - | 105 | 3 |  |
| Hap II | Improved variety | - | - | 1 | - | 3 | - | 20 |
|  | Landrace | 1 | - | - | - | 15 | - |  |
| Hap III | Improved variety | - | - | - | 1 | 3 | - | 13 |
|  | Landrace | 3 | - | - | 1 | 5 | - |  |
| Hap IV | Improved variety | - | - | - | - | 1 | - | 7 |
|  | Landrace | 5 | - | - | - | 1 | - |  |
