# Supplementary figures and images for: Genome-Wide Association Analysis Identifies Resistance Loci for Bacterial Leaf Streak Resistance in Rice (Oryza sativa L.)
Source: Plants (Basel). 2020 Nov 29;9(12):1673. doi: 10.3390/plants9121673 (PMC7761455; doi:10.3390/plants9121673)

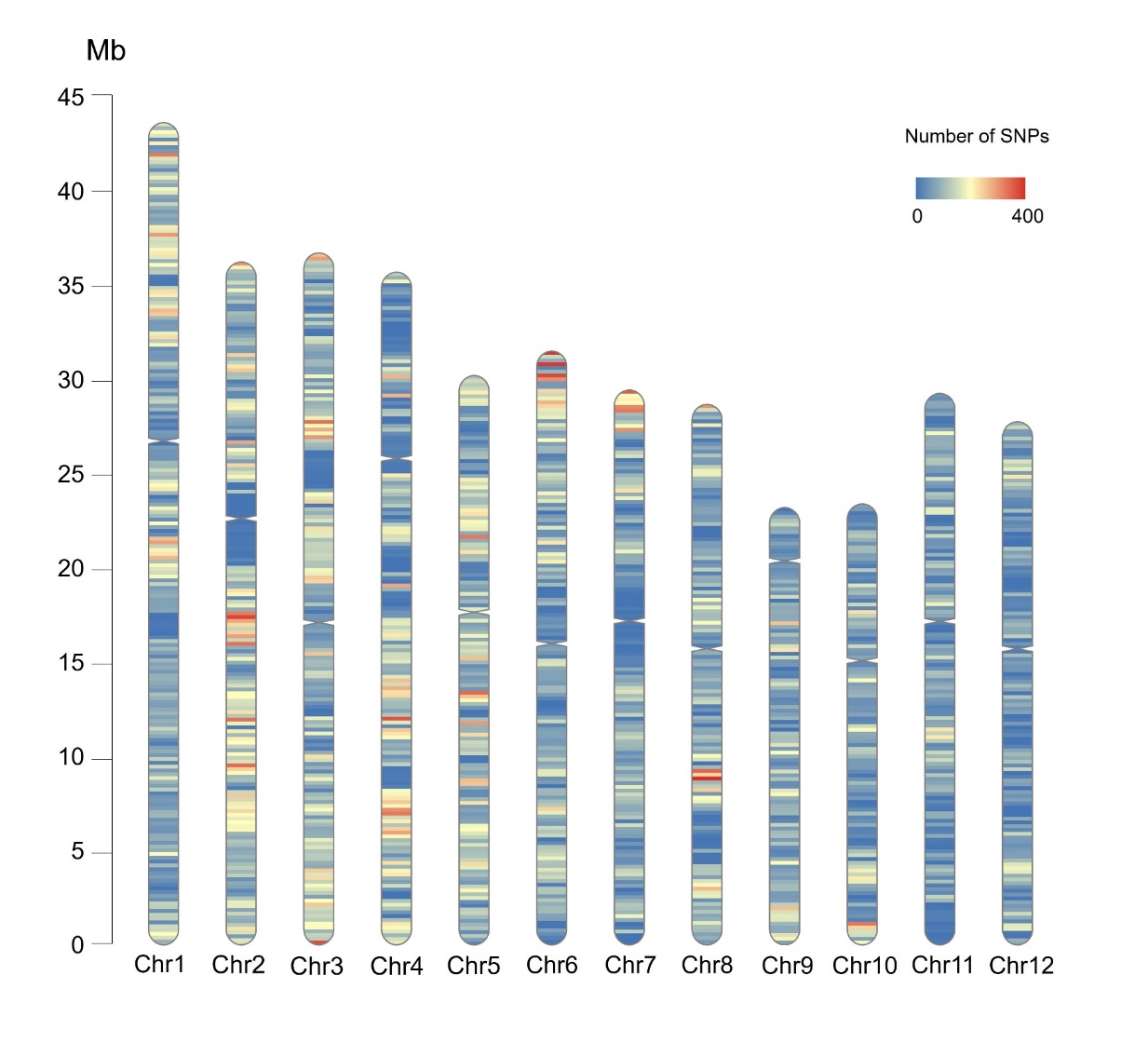


**Figure S3**. Density and distribution of SNPs throughout 12 rice chromosomes

Supplement: Supplementary file 1 [file plants-09-01673-s001.zip › Supplementary/Fig.S3-SNP density.docx]

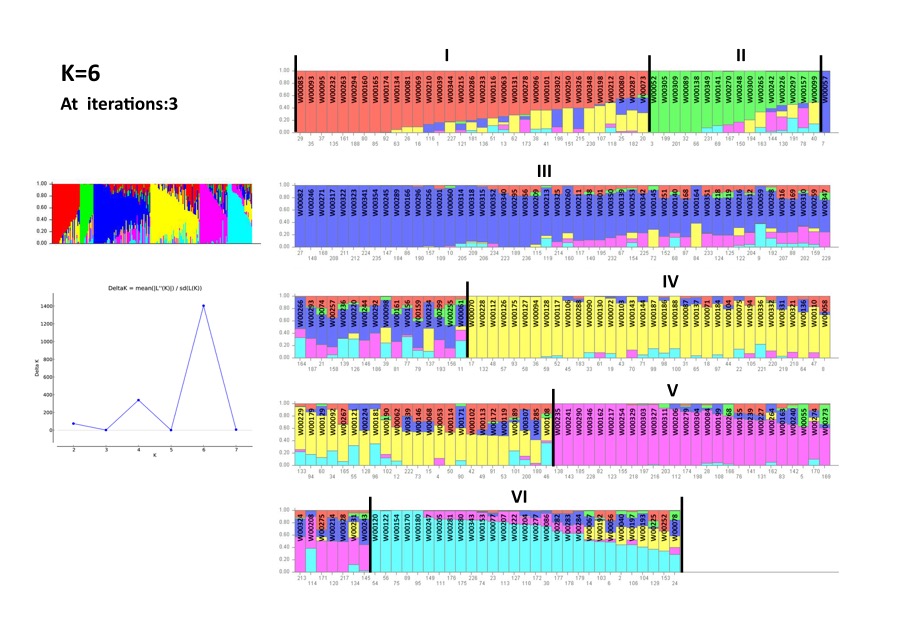


**Figure S4.** Population structure at K = 6 for 236 rice accessions.

Supplement: Supplementary file 1 [file plants-09-01673-s001.zip › Supplementary/Fig.S4-STRUCTURE .docx]
